# Colony Collapse Disorder (CCD) in Honey BeesCaused by EMF Radiation

**DOI:** 10.6026/97320630014521

**Published:** 2018-12-21

**Authors:** Sundar Santhosh Kumar

**Affiliations:** 1 Department of Computer Science, Alagappa University, Karaikudi, Tamil Nadu, India - 630 003

**Keywords:** Electromagnetic radiation colony collapse disorder., cell towers, honey bees, colony collapse disorder.

## Abstract

Honey bees are one of the treasures in the world. An increase of waveform communication leads to good information exchange of mankind. In the biological view, it causes a lot of side effects and lifestyle changes in other living organisms. The drastic changes are causing the natural imbalance in the ecosystem and become a global issue. There are significant reasons for bee colony collapse disorder (CCD) like pesticides, disease and climate change. Recent studies reveal that a cell phone tower and mobile phone handset are also causing side effects to honey bees due to radiation emission. Most of the researchers concentrated on biological and behavioral changes in a honey bee due to radiation effects. For that, the real-time radiation levels have experimented but the different technical perspectives such as radiation emission levels, handset radiation emission measures and multi-sources of radiation are needed to be considered during research. This study aimed to provide possible research extensions of colony collapse disordercaused by cell tower and mobile handsets.

## Background

**Figure 1:**
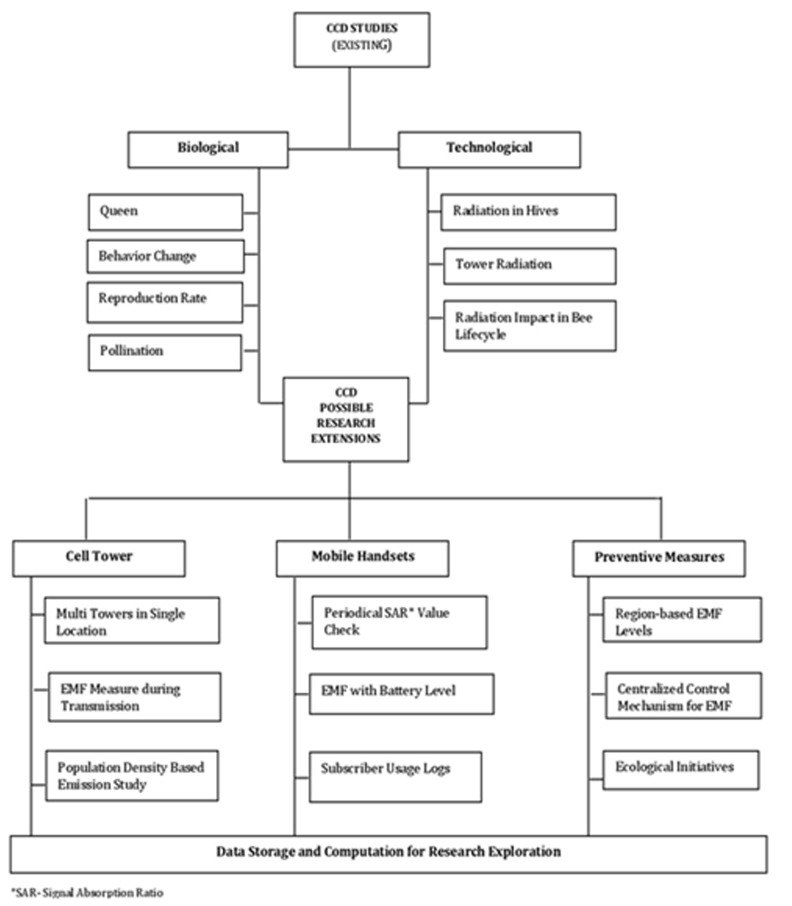
Diagramatic Representation of CCD Research Perspectives

Honey bees are small insects which play a vital role in agriculture. Honeybees are essential partners of pollination for successful yield in agriculture. Recent declines in honey bee populations and increasing demand for insect-pollinated crops raise concerns about pollinator shortages. It happens due to pesticides, monocultural crop practices etc. 1. The recent study reveals that another potential cause for bee losses are electromagnetic fields. The sudden growth in the telecommunication sector leads to the manifold increase of mobile phone and exponential installation of cell towers across the nations. The sudden loss of honey bees in a colony is called colony collapse disorder (CCD) 2. It is a syndrome with no adult bees in with a queen in a colony. It is said to be a dead colony. The cellular service providers and governing bodies confirming that there are no side effects due to cell tower radiation and cell phones. According to plant tree foundation (PTF) in America, there is no real evidence that honeybees rely on the electromagnetic field to navigate, and many apiaries that are still experiencing losses are in rural areas where cell phone service is spotty or absent 3. Some of the researchers revealed that there is standard evidence that the EMF radiation cause damage in honey bees. A large number of studies have been performed over the last two decades to assess whether cell towers and mobile phones pose a potential health risk or not. In another view WHO conducted a formal risk assessment of all studied health outcomes from radiofrequency field's exposure by 2016 whereas WHO (2010) reported there was no standing evidence for causing health defects in the ecosystem by EMF radiation 4,5. In the year 2017 Olgasheean, former international civil servant, brain-tumor survivor and electro-sensitive individual from World Health Organization who initiated a project for establishing a globalized standard for wireless communication which comprises mobile device standards, radio wave, and microwave emission limitations 1 [6]. The important agenda for the mission is to protect the world population from harmful microwave radiations. The WHO has given the green light to governments, regulatory bodies, service providers, and healthcare agencies around the world to consider radiation emission issues. Based on that the Department of Telecom (DOT) India in 2017 launched a web portal called “Tarang Sanchar" that allows people to know radiation emission levels of cell towers across the country 7, 8.The problem is whether honey bees affected by radiation in permissible levels orwhen high-level radiation occurs. These areas should be explored in both biological and technical perspectives. There are significant studies done in the biological perspective hence the limited number of works done in technical perspective. For that, the present study investigates existing experimental studies and analyzes to find possible research paths (Figure 1).

## Evidential Study:

The existing experimental studies contributed much more in CCD research area. The overall work investigation is classified in three major categories namely honey bee radiation study in hives, the impact of the life cycle of honey bees caused by EMF radiation, CCD caused by cell towers.

## Study of CCD caused by Radiation in Hives:

The first criteria are well explored and the studies confirmed the radiation effects arepossible for CCD due to mobile handsets. The waggling dance, foraging, and navigation behavior of honey bees are destructed due to radiation emission of mobile phones. Is electromagnetism one of the causes of the CCD? A work plan for testing this hypothesis, Marie-Claire Cammaerts (2017) 9. The experimental study made in Belgium. The honey boxes with and without mobile phones have experimented in radiation exposure. The health condition and behavioral changes in honey bees are predicted during experimentation. The study has shown that the honey bees hesitated to enter into the hive where mobile handset is placed inside. The bee felt discomfort and given alert sound about the indication of danger. The count of bee entered in another entrance (without mobile handset) is comparatively high and no behavioral change was recorded. Electromagnetic Radiation (EMR) Clashes with Honey Bees, Sainudeen Sahib.S (2011) done an experimental study with six honey hives 10. The selected hives with mobile handsets and results shown the strong destruction of navigational skills in worker bees and colony collapsed due to high radiation emission. The study did notcover any distance measures between cell towers, mobile handsets, and hives.

Study of radiation influences in a life cycle of honey bees: Most of the studies focused on behavioral change in honey bees due to EMF radiation. Some significant contributions are in queen and its hatching measures. Changes in honeybee behavior and biology under the influence of cell phone radiations,VedParkash Sharma and Neelima R. Kumar (2010), made a significant study of honey bees during radiation exposure and revealed remarkable outcomes of their work 11. The results have shown that the life cycle of the honey bee is affected by electromagnetic radiation exposure. Influence of cell phone radiations on aphismellifera semen Kumar, Neelima r., tarunaVerma and anudeep (2012), examined male honey bee to prove the impact of electromagnetic radiation in their semen 12. This work revealed that the genetic disorder in a brood may possible due to EMF by mobile phone and cell towers. The test made with mobile handsets kept inside the hives. The Effect of Cell Phone Radiations on the Life Cycle of Honeybees,Nashaat El Halabi, Roger Achkar, Gaby AbouHaidar (2014) made a study in a different view. They measured variation in sound by honey bees during radiation emission. The sound emitted by bees is recorded in various conditions and resulted that the honey bees are disturbed by mobile radiation and they acted with different behavior during radiation 13.

## Study of CCD caused by cell towers:

Only a few of the studies are done about EMFradiation in cell towers. The cell tower and multiple cell towers in the same location can have the possibility of colony collapse. The mobile phone with respect to its density and battery level also be considered. For that SAR and Connect values will be used as the measuring parameters. Effect of electromagnetic radiations on brooding, honey production and foraging behavior of European honey bees, Pramod mall and YogeshKumar (2014) made an experimental study and resulted that there is an insignificant change in honey bee colony by electromagnetic radiation 14. The three-phenomenon taken into the study is a colony under cell phone tower - The cell phone tower radiation emission is zero degree at this point. Therefore, the radiation must be very less and it will not create any major risks. For example, may birds are having to construct their nest in cell phone towers. The second experimentation was done with mobile phone kept in a colony. Mobile phones will not cause any biological damages in a colony is acceptable but the mobile phone radiation level increases during communication time only. Hence the test did not include that criterion. Effect of electromagnetic radiation of cell phone tower on the foraging behavior of Asiatic honey bee, Apiscerana F. (Hymenopteran: Apidae), RituRanjanTaye, Mukul Kumar Deka, Ataur Rahman and ManhaBathari (2017) made an experimental study about CCD caused by cell phone towers 15. The test is carried in different distance levels from cell towers with the colony. They calculated foraging behavior, in and out the count of bees and honey collected by each honey bee to the nest. This experimental study reveals that there is a slight loss of all activities in the colony where is closer to the radiation level. The foraging behavior may possibly have affected due to cell tower radiation.

## Research extenstions in CCD:

Based on the several experimental studies the following possible research areas can be explored in technological view.

## Radiation Emission of Cell Towers and CCD

Most of the studies concentrated in EMF radiation of Mobile handsets with CCD. The EMF emission of cell towers must be considered as a serious problem and lot research is needed in that area. Multiple cell towers installed in the same location is also considered. Whereas each tower may emit radiation in permissible limits but overall radiation should be measured and CCD issue to be explored. Cell towers with high-density population produce high radiation in both cell towers and mobile phones. Radiation Emission of Mobile handsets and Battery power The mobile handset with less battery power and low signal range causes high radiation exposure. The distinct tower location areas, especially in forest areas must be explored. Experimentation should be done periodically during radiation and some studies done only with mobile handsets nearby hives.

## Preventive Mechanisms

The radiation shielded honey boxes may use to protect the hives from high radiation exposure. The other preventive mechanisms are also being considered like planting Trees and gardens. The less number of trees and plants insists bees to collect honey from long distances. Hence it is affected by environmental factors and failed to return their hives. If trees are available in frequent distances, then it can be avoided. The urban areas can be gardened in public areas like park etc.

## Data Storage and Computation

Daniel Favre (2017), an Independent researcher revealed that the experimental data about CCD and honey bees research with respect to EMF must be shared and contributed to the researchers for further exploration of research in that area. The experimental data can be used as a data set to predict accurate results using computation techniques. Another important point is experimental data are the strong shreds of evidence for radiation occurrence so that the impact of radiation can be known to the society. The data set can be stored in public storage medium as online databases or in cloud storage. So, that it is available and accessible globally.

## Conclusion

The present investigations were undertaken significant studies in CCD and radiation effects. The perspective of this proposed study to enable researchers to extend their research work from a technological point of view. The existing studies show both negative and non-negative impacts of radiation. whereas the nonnegative studies need to confine that there is standard proven evidence for the destruction of bee colony due to radiation. When the studies have supporting data with technological evidence then it can be considered as a blueprint for researchers and social workers to insist the authorities to standardize the regulations to control and maintain the radiation emissions by cell towers and mobile handsets.

## References

[1] Odemer R, Odemer F (2018). BioRxiv..

[2] Gil-Lebrero S (2017). Sensors Basel.

[3] http://www.pattfoundation.org/.

[4] https://www.who.int.

[5] Hardell L (2017). Int J Oncol..

[6] https://olgasheean.com/.

[7] http://www.dot.gov.in/.

[8] http://www.tarang.website/home.

[9] Marie-Claire C (2017). J Behav.

[10] Sainudeen SS (2011). Int Jour. Env. Sci..

[11] Sharma VP (2010). Current Science.

[12] Neelima RK (2012). J. Global Biosciences.

[12] El Halabi N (2014). Proceedings of the Mediterranean Electrotechnical Conference MELECON.

[12] Mall P, Kumar Y (2014). African J of Agri Research.

[15] Taye RR (2017). J Ent Zoo Studies.

